# Diversity of* Mycobacterium tuberculosis* Complex from Cattle Lymph Nodes in Eastern Cape Province

**DOI:** 10.1155/2018/3683801

**Published:** 2018-04-10

**Authors:** Nolwazi Londiwe Bhembe, Godfred Ngu Tanih, Lesley-Anne Caine, Abongile Pekana, Patrick Govender, Uchechukwu Uchechukwu Nwodo, Anthony Ifeayin Okoh, Leonard Vuyani Mabinya, Ezekiel Green

**Affiliations:** ^1^SAMRC Microbial Water Quality Monitoring Centre, University of Fort Hare, Alice 5700, South Africa; ^2^Applied and Environmental Microbiology Research Group, Department of Biochemistry and Microbiology, University of Fort Hare, Alice 5700, South Africa; ^3^Department of Biotechnology and Food Technology, Faculty of Science, University of Johannesburg, Doornfontein 2028, South Africa; ^4^Department of Biochemistry, School of Life Sciences, University of KwaZulu-Natal, Private Bag X54001, Durban 4000, South Africa

## Abstract

Tuberculosis (TB) remains a major health challenge in South Africa and the condition in humans has been well researched and documented. However, investigations on the circulating* Mycobacterium tuberculosis* complex (MTBC) strains from cattle in the Eastern Cape Province of South Africa are insufficient. This study delineated the diversity of MTBC isolates from cows' lymph nodes. A total of 162 MTBC isolates, collected over a one-year period from cattle lymph nodes from two abattoirs, were submitted to spoligotyping and 12 MIRU-VNTR typing. The spoligotyping results were matched with isolates in the universal spoligotyping database (SITVIT2). Our study identified 27 spoligotype patterns, with 10 shared types assigned to five lineages: the East-Asian (Beijing) was predominant, 17.9%, and East-Asian (Microti) and Latin-American-Mediterranean S were the least detected with 0.6%. Spoligotyping showed a higher clustering rate of 82.1%, with the lowest being the Hunter-Gaston Diversity Index (HGDI) of 0.485; 12 MIRU-VNTR resulted in a clustering rate of 64.8%, showing a higher HGDI of 0.671. The results of this study show a high diversity of MTBC strains in the Eastern Cape Province and clustering rate, which indicates ongoing transmission in the province.

## 1. Introduction

South Africa ranks the third highest globally with the number of predictable leading (undiagnosed dynamic TB) cases [1]. In 2013, the country reported TB as the predominant cause of death (over 40,542 deaths in that year) [2]. In 2014, 834 cases/100,000 of the population were reported [3]. In 2015, 454,000 instances of active TB were reported from a population of 54 million [4]. Eastern Cape (EC) Province has the worst TB situation, despite the TB control programmes used in the province [5]. In 2015, 692 cases/100,000 inhabitants were reported in this province [4]. The high TB prevalence is ascribed currently to HIV coinfection, poverty, and TB drug resistance [5]. However, dairy farming is a common practice in this province, with farms supplying large amounts of unpasteurized milk to urban and rural areas, consumed by individuals to supplement their diets. We argue that this consumption of unpasteurized milk poses a serious public health problem due to the potential of transmitting MTBC (especially* M. bovis*) to humans [6].

Bovine tuberculosis (bTB) is a well-known zoonotic infection with an extensive array of hosts, including humans [7]. The prevalence of bTB is unknown in South Africa, although it is suspected to be the cause of commercial farming losses. BTB can infect individuals and animals in developing and nonindustrialized countries; in fact,* M. bovis* is liable for about 5–10% of all TB occurrences and 30% of epidemics in juvenile human TB patients [8]. Appropriate analysis of bTB is vital for the proper control and prevention of MTBC strain transmission to both humans and animals [9]. It is essential to know the quantity and type of strains disseminated in the field, and therefore genotyping is necessary in the molecular epidemiology of MTBC for appropriate monitoring of prevalent strains and strain families that are overrepresented [10].

The understanding of TB transmission patterns and the hereditary diversity of MTBC strains has been greatly improved by the introduction of molecular epidemiology [11, 12]. The genotyping methods used include IS*6110 *restriction fragment length polymorphism (IS*6110*-RFLP), spacer oligonucleotide typing (spoligotyping), and variable-number tandem repeats of mycobacterial interspersed repetitive units (MIRU-VNTR). These have been proven to be effective tools for bTB epidemiological investigations and infection control [13]. IS*6110*-RFLP was once a gold-standard method but it requires large quantities of DNA and cannot differentiate organisms with 5 IS*6110* copies. The spoligotyping and MIRU-VNTR genotyping methods were established [14]. Comparing the three methods, MIRU-VNTR and spoligotyping have the advantage of being more rapid and appropriate for all MTBC isolates, as well as strains with a few IS*6110* copies [15, 16].

Initially, the VNTR typing systems for MTBC used a limited set of loci [15, 17, 18], which were not adequately discriminatory [19]. Broad sets of VNTR loci have been reported to be better suited for dependable genotyping and molecular investigations of MTBC [14, 16]; this system is based on 12 loci. The application of 12 locus-based MIRU-VNTR and spoligotyping techniques has been reported to provide high satisfactory discrimination for large-scale and local outbreak investigations [20, 21]. Spoligotyping has resulted in the construction of a well-used international database that assigns an identity to a given strain and documents the global phylogeography of MTBC strains [22]. The occurrence and transmission of MTBC strains vary in countries and regions within the same country [23], and proper identification of MTBC strain families in specified geographical vicinities is significant for epidemiological investigations.

In South Africa, studies on mostly genotyping assays have been carried out in a few provinces, with high reported multidrug-resistant TB strains (MDRs); these are in Western Cape [24–27], Gauteng [28–31], and KwaZulu-Natal [32, 33]. This study was designed to determine the MTBC strains' dissemination from cattle lymph nodes in Eastern Cape Province of South Africa using the MIRU-VNTR (original 12 loci) and spoligotyping.

## 2. Method

### 2.1. Sample Collection, Handling, and Molecular Identification

376 lymph nodes showing lesions were collected from a total of 14,950 slaughtered animals in two abattoirs found in Eastern Cape Province (Chris Hani and Buffalo City municipalities) for a year as earlier reported in our larger study by Bhembe et al. [34]. Samples were decontaminated before culture following the protocol reported by Bhembe et al. [34]. Each sample was cultured on three different Löwenstein-Jensen (LJ) slants. The first two slants (supplemented with either glycerol or pyruvate) were cultured in preparation for spoligotyping and MIRU-VNTR typing. All three LJ slants (BD Biosciences, Sparks, MD, USA) were inoculated with 300 *μ*l of the decontaminated sample.

Slants were incubated (placed sidelong) overnight at 37°C before being turned upright. The slants were ventilated every 3 days for three weeks and then every week for 6 to 8 weeks depending on the growth perceived. The slants were then kept at 4°C to preserve the organisms.

The growth of MTBC was identified by the formation of gritty white/cream colonies on the slants. Deoxyribonucleic acid (DNA) was extracted from the slants with colony growth, following the method outlined by Berg et al. [35]. DNA concentration was obtained by measuring the absorbance using a Thermo Spectronic, BioMate 3 Spectrophotometer (Rochester, New York, USA) and confirmation of MTBC was done using PCR as outlined by Bhembe et al. [34]. The study was authorized by the University of Fort Hare Research Ethics Committee, and an ethical clearance was issued (REC-270710-028-RA).

### 2.2. Molecular Genotyping

#### 2.2.1. Spoligotyping

Spoligotyping was carried out using a spoligotype kit (Isogen, Bioscience B.V., Utrecht, Netherlands) and PCR amplification, hybridization of amplified products, and detection and interpretation of results and recording of signals were executed as outlined by Kamerbeek et al. [36].

#### 2.2.2. MIRU-VNTR

The isolates were further evaluated using 12-locus MIRU-VNTR typing. Amplification of MIRU loci was achieved individually with the primers specific for the flaking regions of each locus [13]. The PCR amplification mixture for each reaction contained 1x master mix (0.05 U/*μ*l) Taq polymerase, 0.2 mM each of the deoxynucleotide triphosphates (New England Biolabs, Inc., Hitchin, UK), 2 mM MgCl_2_, 2 *μ*M of each primer pair, and 2 *μ*l of DNA, and nuclease-free water was added to make a total volume of 25 *μ*l.

Amplification was achieved using a MyCycler™ Thermal Cycler (Bio-Rad, Cape Town, South Africa) with 1 cycle of denaturing at 95°C for 15 minutes, followed by 38 cycles of 1 minute at 95°C and 1 minute at 72°C, followed by 1 cycle of final extension at 72°C for 10 minutes. The positive (H37Rv strain) and negative (sterile nuclease-free water) controls were added to the amplification. The amplicons were segregated on 3% agarose gel (Whitehead Scientific (Pty) Ltd., Cape Town, South Africa) using 100 bp ladder (New England Biolabs Inc., Hitchin, UK) as the size marker.

### 2.3. Data Analysis

All biotype results were captured into a Microsoft Excel sheet. The octal format and binary code for spoligotyping patterns and 12-locus MIRU-VNTR profiles were compared to all of those available in the SITVIT2 database as reported by Demay et al. [37]. The SITVIT WEB of Pasteur Institute of Guadeloupe was used to compare the spoligotyping data to more than 75,000 clinical isolates matching to 153 countries [38] and assign SIT numbers or orphan status to uploaded strains [37]. The SIT number was matched to strains that had identical patterns to patient isolates available on the database and isolates that did not have identical profiles were considered as orphan strains. The updated SpolDB4 was used to assign the lineages to the strain level. Orphan strains were further assigned TB lineages using the TB-lineage, following the application of the presence and absence of specific spacers rule using the SpolDB3 model [39]. This model assigns genotypes into the seven lineages classified as East-Asian, known as Beijing, Euro-American, and East-African Indian and ancestral (Western African 1 and Western African 2, representing* M. africanum*,* M. bovis*, and Indo-Oceanic) MTBC strains [40].

Phylogenetic trees were constructed based on spoligotyping, MIRU-VNTR, or their combined patterns using the MIRU-VNTRplus site (http://www.miru-vntrplus.org/MIRU/index.faces) [41]. Jaccard's constant was employed to determine the distance matrix and neighbour-joining (NJ) clustering algorithms [13]. The MIRU-VNTRplus Database (http://www.miru-vntrplus.org) was then used to match the 12 MIRU-VNTR patterns, calculated for each of the loci using the Levenshtein algorithm (edit distance).

### 2.4. Statistical Analysis

The method of Hunter and Gaston [42] was used to calculate the Hunter-Gaston Index defining each MIRU-VNTR locus of the strains. The subsequent equation was used to obtain the diversity index, where *N* is the total number of strains in the sample populace for a known locus, *S* is the total number of different repeat unit values identified for the locus, and *nj* is the number of isolates having the *j* value:

(1) $$\begin{array}{c} HGDI=1-\frac{1}{N\left(N-1\right)}\overset{S}{\underset{J-1}{\sum }}nj\left(\;nj-1\;\right). \end{array}$$

The confidence interval, which is the accuracy of the diversity index articulated as 95% (upper and lower precincts), was also calculated with the Hunter-Gaston Index accessible from (http://www.hpa-bioinformatics.org.uk/cgi-bin/DICI/DICI.pl).

## 3. Results

**Figure 1:**
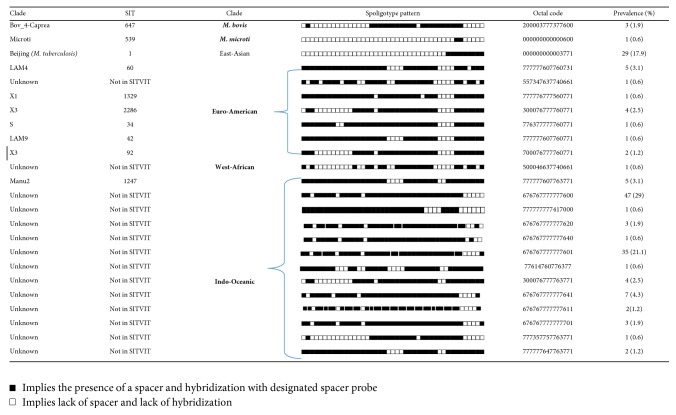
Different spoligotype international types detected from slaughtered cattle lymph nodes in Eastern Cape Province, South Africa.

Ten SITs belonging to five lineages were found in this study, with the lineages being the East-Asian (Beijing),* M. microti*, Euro-American, Indo-Oceanic, and* M. bovis*. The less frequent lineage was the Euro-American with three different clades, namely, LAM9, S, and X1 (0.6% each), as shown in Figure 1. The East-Asian (Beijing) was the predominant lineage with 29 among 162 isolates (17.9%). In this study, we found 110 (67.9%) orphans (isolates with unknown families) and 52 (32.1%) isolates from 10 spoligotype international types (SITs). The orphans were further demarcated in the SITVIT2 database following the SpolDB outlined rules (Figure 1). The 110 orphans after being further defined in the SITVIT2 constituted three lineages: Euro-American (1/110), Indo-Oceanic (108/110), and West-African 2 (1/110).

**Figure 2:**
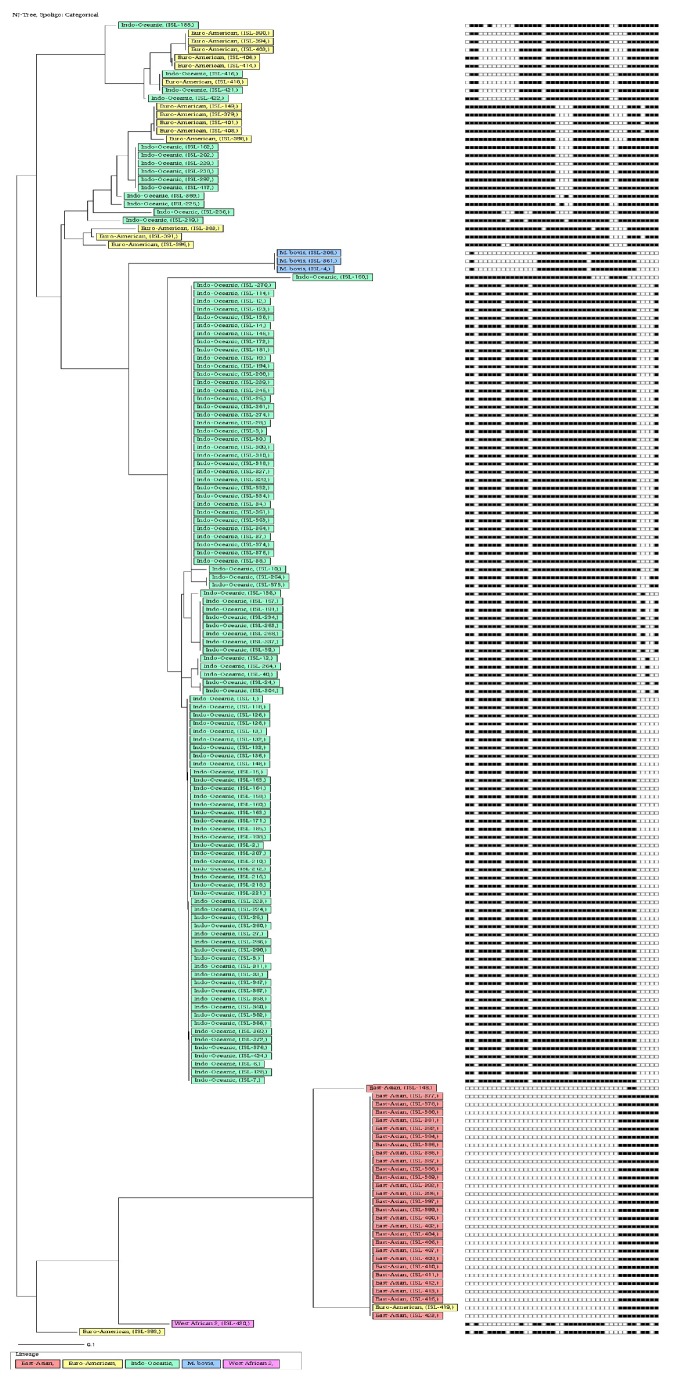
Relationship of only spoligotyping analysis of DNA isolated from cattle lymph nodes.

All three methods applied gave different clusters and clustering rates when phylogenetic trees were drawn. Spoligotyping alone exhibited 13 clusters (Figure 2) with an 82.1% clustering rate (Table 1) and VNTR alone exhibited 8 clusters (Figure 4) with a clustering rate of 4.8%. When spoligotyping and MIRU-VNTR analysis were combined, 11 clusters were revealed (Figure 5), with a 33.7% clustering rate (Table 1).

**Figure 3:**
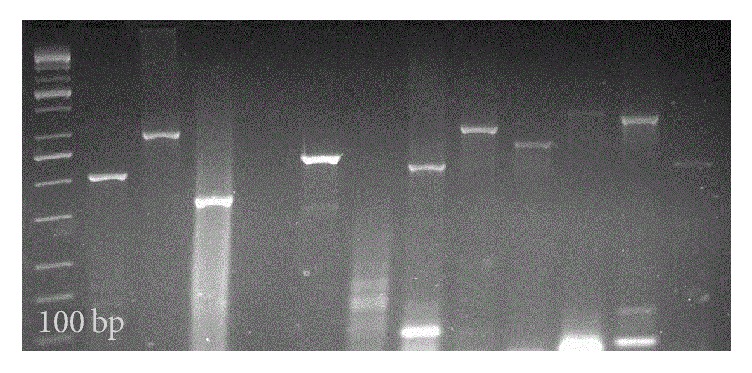
*Amplification products of the 12 alleles of MIRU-VNTR from a PCR analysis for a Bov_4-caprae strain for M. bovis (ISL-205).* Lane 1: 100 bp DNA ladder (Thermo Fisher Scientific), lane 2: allele 580, lane 3: allele 2996, lane 4: allele 802, lane 5: allele 3192, lane 6: allele 2687, lane 7: allele 980, lane 8: allele 2531, lane 9: allele 2058, lane 10: allele 154, lane 11: allele 4348, lane 12: allele 1644, and lane 13: allele 3007.

**Figure 4:**
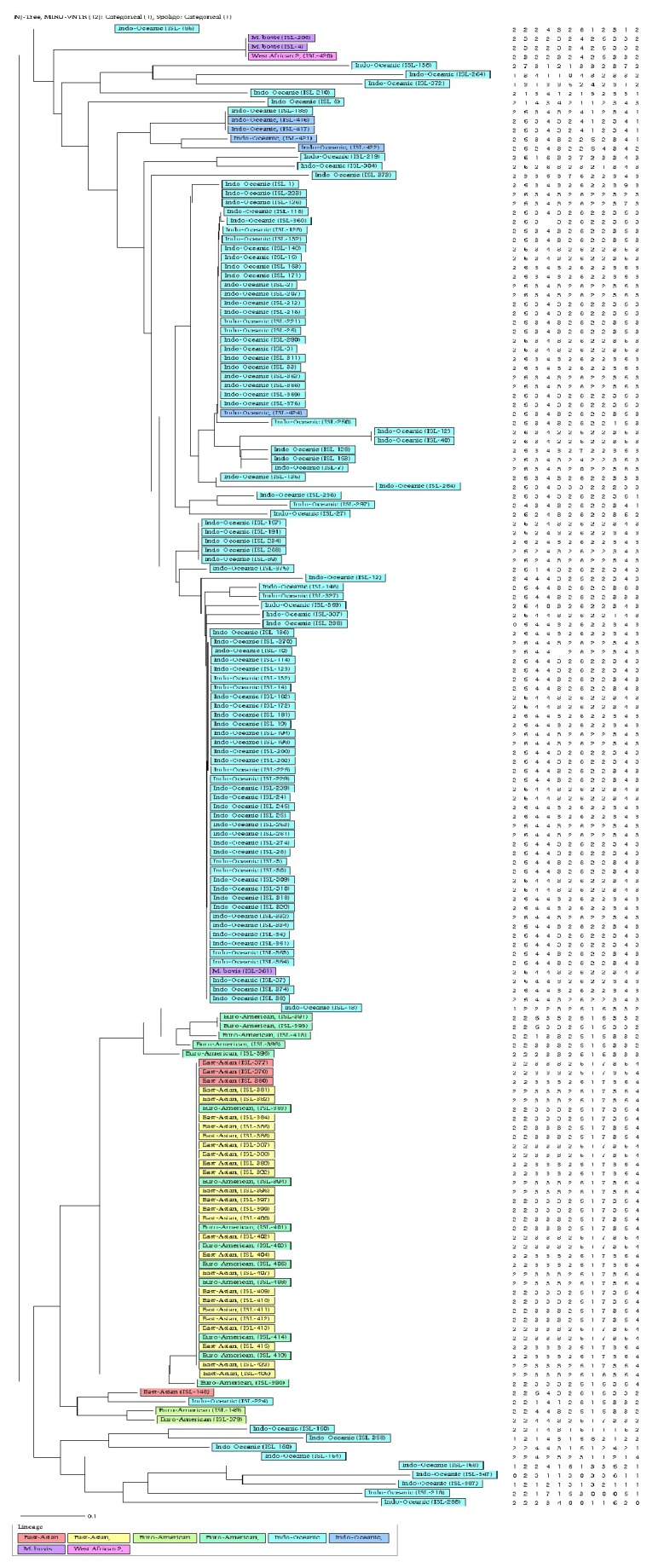
Relationship of MIRU-VNTR analysis of DNA isolated from cattle lymph nodes.

**Figure 5:**
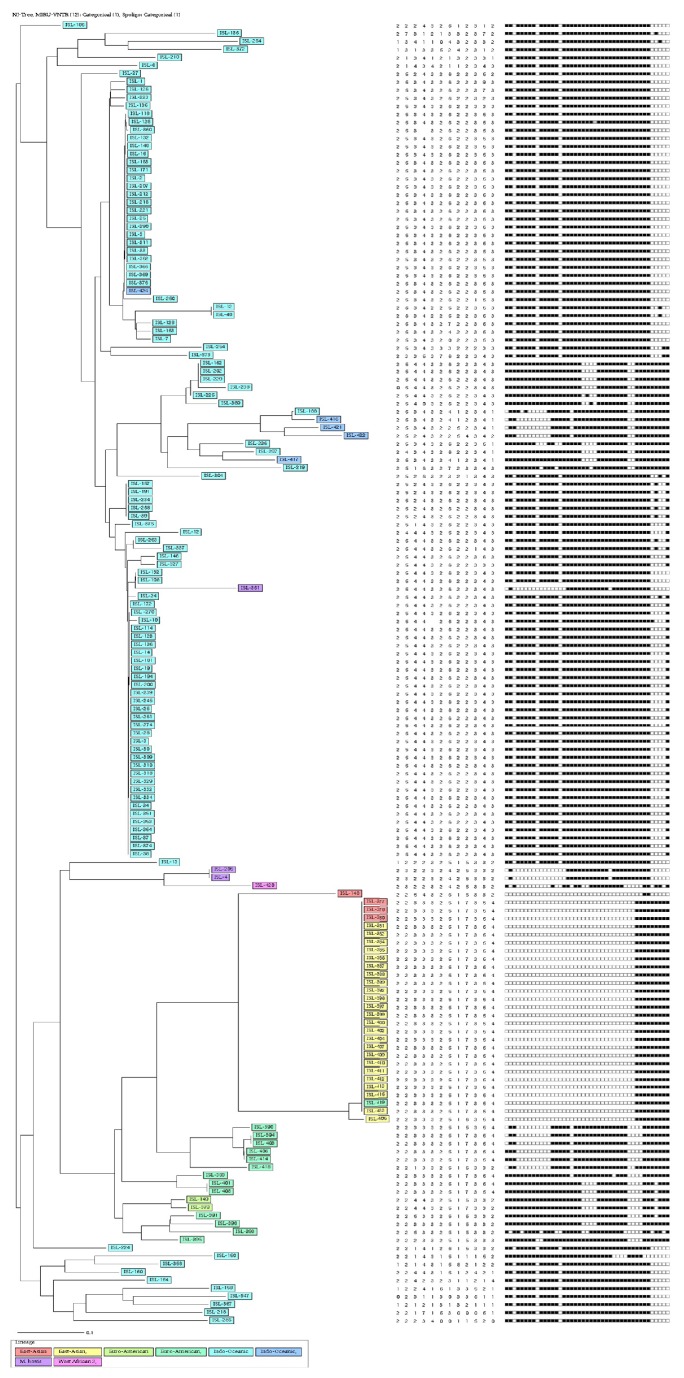
*Association of spoligotypes of DNA isolated from cattle lymph nodes collected in Eastern Cape Province (data for both spoligotype patterns and MIRU-VNTR analysis).* Alignments were engendered with the MIRU-VNTRplus web application (http://www.miru-vntrplus.org/MIRU/index.faces).

A confirmation of the 162 amplification products of the 12 alleles of a PCR analysis MIRU-VNTR for a Bov_4 caprea strain is shown in Figure 3.

The allelic diversity of the 12 MIRU-VNTR loci observed in this study ranged from 0.118 to 0.671, with MIRU 31 being the highest occurring locus with the highest diversity index of 0.671 (*h* > 0.6) and MIRU 1 having the lowest diversity index of 0.118 (*h* < 0.3) (Table 2).

HGDI is Hunter-Gaston Diversity Index; PD means poorly discriminatory (*h* < 0.3); HD means highly discriminatory (*h* > 0.6); MD means moderately discriminatory (0.3 ≤ *h* ≤ 0.6) [41, 42].

Single methods showed a lower discriminatory power than the combined method. Spoligotyping alone had the lowest diversity index (DI) (DI = 0.485) and a confidence interval of 0.449–0.520. MIRU-VNTR alone (DI = 0.671) had a confidence interval of 0.629–0.712 at MIRU 31. Combining the two approaches of spoligotyping and MIRU-VNTR gave the highest (DI = 0.676) discriminative power and a confidence interval of 0.635–0.718 at MIRU 31 (Table 1). Combining the two methods gave a higher discriminatory power than when the techniques were used discretely.

## 4. Discussion

South Africa is one of the countries with the highest burden of TB [43], and therefore it is important for South Africa to detect predominant strains to observe alterations in strain conformation within populations and to document the epidemiology of the infection. The nation's MTBC diversity strains have been defined in various provinces including the Free State [23, 24, 26, 30–33, 44, 45]. Eastern Cape Province, which is the third largest province of South Africa with approximately 63% of the province's population living in rural areas, has most data on human sputum samples [46]. However, Silaigwana et al. [6] reported high prevalence of MDR-TB in cattle in the Nkonkobe municipality of Eastern Cape Province. This indicates the increased risk of MTBC transmission between animals and also to humans.

This province has the second worst burden of poverty in South Africa, with a more than 55% rate of unemployment, which results in a high percentage of the population depending on subsistence farming [6]. Small stock farmers live in a nearby vicinity to their livestock to minimise the risk of theft. This could be a risk of transmitting MTBC strains from humans to animals and vice versa. Most people in the province consume unpasteurized milk from small-scale farmers to supplement their diets due to its low cost [6]. This could be another possibility of transmitting TB strains from cattle to humans and other animals (calves). Hence, it is significant to screen for MTBC in unpasteurized milk before human consumption. This study reports strain diversity from cattle lymph node samples in Eastern Cape Province for the first time.

The two techniques used in this study, namely, MIRU-VNTR typing and spoligotyping, make a good combination for detecting and genotyping the MTBC, including* M. bovis* [14, 47, 48], which has been reported to have few IS*6110* or DR copy numbers [19].

Our results of 162 MTBC isolates show a high diversity of strains representing seven main lineages, namely, Bov_4-caprea (SIT 647), LAM (SIT 60), MANU (SIT 1247), Beijing (SIT 1),* M. microti* (SIT 539), S (SIT 34), and X (SIT 1329, 2286, and 92). There were also several orphan strains assigned to their most appropriate lineage and sublineage using the TB-insight database, which classified 110 strains into 3 lineages (1 Euro-American, 108 Indo-Oceanic, and 1 West-African 2). In the study, the Indo-Oceanic lineage, 69.8% (113/162), was more predominant. We were not able to further classify the Indo-Oceanic strains to their subsequent clades, East African-India (EAI) and MANU [49, 50]. We believe that our study has identified the Indo-Oceanic lineage in Eastern Cape Province for the first time in cattle, while the Beijing, LAM, and T families have been reported to be predominant by Said et al. [46]. The MANU and EAI families (from Indo-Oceanic) have been reported in 5 out of 9 provinces of the country from humans, namely, Gauteng [29], Free State [23], North-West, Limpopo [22], and Western Cape [51]. These families are prevalent in East, Middle, and Central Asia [22, 52].

The most frequently identified strain families in Africa are the families H, LAM, and T [22, 26, 53–56], which were not prevalent in our study, with only 2 being identified (0, 3.71%, and 0, resp.). Although these families have been identified in the African continent, their proportions are not consistent throughout the region and, in some countries, a few predominant strain families fuel the TB spread [53, 57]. These results of this study conducted in South Africa were expected because the strains for this study were isolated from cattle lymph nodes, not the human TB patients investigated in most studies. The underrepresentation of the three predominant families in Africa including South Africa could be the result of the lack of genotyping studies investigating strains isolated from cattle lymph nodes in South Africa. Additional analysis of strain spoligotypes isolated from cattle is mandatory to examine strains in the different provinces of South Africa.

In our study, we noted 1.9% prevalence of the* M. bovis *4 subspecies-*caprae* (SIT 647), known as* M. caprae* strain. This strain has caprine herds as the primary host; bovine herds, however, as well as humans can also be hosts of this pathogen [58]. The* M. bovis* species was first reported in South Africa in 1880 and was alleged to have arrived with European settlers [59]. The first case of bovine TB in South Africa was reported in Eastern Cape Province isolated from the greater Kudu [60]. The strain has been reported in other countries such as Australia, Czech Republic, Sweden, and Ukraine [36]. The Microti (SIT 539) strain family associated with the East-Asian lineage was also isolated from our study (0.6%). We did not expect to isolate* M. microti* from cattle (a domestic animal) because it is known to be prevalent in wild rodents, and this could indicate transmission from either wild rodents or humans who were infected with this strain. We did not find any previous finding of* M. microti *from South African cattle, although it has been reported from Great Britain, USA, Switzerland, and Germany in llamas, humans, cats, and ferrets [19, 36].

The Beijing family (SIT1), with a distribution of 17.9% (29/162), was the most prevalent family identified. The strain is known to be associated with drug resistance [26, 61–63] and there are several theories that discuss the worldwide distribution of this strain due to its selective benefit over other clinical isolates in causing infections [61] in humans. The Beijing strain is the descendant of the* M. tuberculosis* ancestral strain [64]. The strain has been detected in several African countries in domestic animals [65, 66], thus increasing the risk of human infection.

Humans in rural settlements share proximity with cattle; this increases the possibility of spreading the Beijing strain from infected individuals to cattle. Our results substantiate the theory that bovine herds are sensitive to* M. tuberculosis* and the results concur with reports from other studies stating that* M. tuberculosis*, although primarily hosted by human beings, can infect both wild and domestic animals (including cattle) [67–72]. This signifies that bovine TB is of public health importance and there is a lack of published data on TB strains isolated from cattle in South Africa. Silaigwana et al. [6] reported finding MTBC detected from milk in Eastern Cape Province of South Africa, but genotypic studies of the isolated strains were not carried out. To the best of our knowledge, this study has isolated the* M. tuberculosis* Beijing lineage for the first time in South Africa from bovine herds. The lineage, however, has been isolated from cattle samples in other countries [35, 68, 73] including* M. tuberculosis *from cattle in African and Asian countries with animal TB prevalence of 4.7–30% [68, 70, 74]. The Beijing strain infection in cattle has been identified in countries with a high frequency of human TB [75]. This could be another reason for the prevalence of the Beijing strain from cattle, because South Africa had the third highest prevalence of 10.4 million new global TB cases detected in 2015, together with five other countries (India, Indonesia, China, Pakistan, and Nigeria) [76].

MIRU-VNTR carried out on the isolates showed a higher discriminating power (DI = 0.671) than spoligotyping (DI = 0.485). However, the combination of the binary methods improved the discriminatory power (DI = 0.676). The combination of the two techniques reduced the clustering rate to 53.7%, although spoligotyping alone identified a clustering rate of 82.1% and MIRU-VNTR identified a 64.8% clustering rate.

According to Van Soolingen [77], strains with similar clusters (patterns) are most likely to have been newly infected and are a potential target for epidemiological studies to detect the series of transmission. However, it is difficult to understand the ratio of disease caused by the most recent transmission derived directly from a cluster ratio [78], as this can be caused by different factors. For example, even in rural inhabitants, clustering might derive from concurrent recurrence of developed infection from the equivalent basis [79]. The clustering rate observed from this study was (53.7%) lower than the clustering rates of other studies in South Africa [25, 28, 29, 32].

This difference could be due to the fact that previous genotypic studies conducted in South Africa considered transmission from only MDR-TB strains isolated from humans. From this study, we believe that the low clustering rate observed was because the samples used in this study were from cattle, which are slaughtered within a few years of life, as compared to humans who live long lives, increasing the chances of MTBC infection and allowing the disease to progress and even develop to MDR-TB. Our results, however, do have comparable clustering rates to those detected from other African countries [80–82]. We speculate that the high MTBC lineages commoly reported from human specimens observed from cattle lymph nodes in this study may possibly be due to the proximicity shared between the animals and humans who might be having active TB, thus passing the infection to the animals.

## 5. Conclusion

The results from this investigation elucidate the diversity of TB from Eastern Cape Province. This study reports a high level of typical human MTBC lineages in cattle; this could be due to the fact that farmers share proximity with their animals and these lineages could be transmitted from them to the animals through aerosols. The clustering rate observed from the study may possibly indicate the transmission of different strains in the province. The study also shows the effectiveness of the combination of MIRU-VNTR and spoligotyping (high discriminatory power) and it suggests that it can be used to investigate bovine TB samples in South Africa. The strains that were not identified in the spoligotyping international database (orphans) still need to be further investigated.

## Figures and Tables

**Table 1:** The supremacy of spoligotyping and VNTR used exclusively and in combination.

Typing methods	Number of clusters	Number of clustered isolates	Number of unique isolates	Size of clusters	Clustering rate	HGDI^a^
Spoligotyping	13	146	16	2–48	82.1	0.485
12 MIRU-VNTR	8	113	49	2–41	64.8	0.671
Spoligotyping + VNTR	11	98	64	2–49	53.7	0.676

^a^HGDI: Hunter-Gaston Diversity Index; the clusters that were counted were only the outer clusters (clusters that are directly linked to the root).

**Table 2:** MIRU and allelic diversity of the locus investigated.

Allele	Conclusion	Number of alleles	Diversity index	Confidence interval
154	PD	5	0.118	0.051–0.185
560	MD	9	0.558	0.507–0.610
980	MD	9	0.529	0.462–0.596
1644	PD	6	0.163	0.087–0.241
2058	PD	8	0.165	0.087–0.239
2531	HD	9	0.620	0.564–0.676
2687	MD	8	0.541	0.489–0.593
2996	MD	9	0.543	0.469–0.617
3007	PD	8	0.164	0.087–0.241
3192	HD	9	0.671	0.629–0.712
4348	HD	6	0.613	0.551–0.675
802	HD	6	0.644	0.596–0.692

## References

[1] Mahbubani K. (2012). The World Health Organization (WHO). *Global Public Health*.

[2] Statistics South Africa Mortality and causes of death in South Africa. Findings from death notification. http://www.statssa.gov.za.

[3] World Health Organization (WHO) Global Tuberculosis Report. http://www.who.int/tb/publications/global_report/en.

[4] World Health Organization (WHO) Global Tuberculosis Control. http://www.who.int/tb/en/.

[5] Karim S. S. A., Churchyard G. J., Karim Q. A., Lawn S. D. (2009). HIV infection and tuberculosis in South Africa: an urgent need to escalate the public health response. *The Lancet*.

[6] Silaigwana B., Green E., Ndip R. N. (2012). Molecular detection and drug resistance of *Mycobacterium tuberculosis* complex from cattle at a dairy farm in the Nkonkobe region of south africa: A pilot study. *International Journal of Environmental Research and Public Health*.

[7] O'Reilly L. M., Daborn C. J. (1995). The epidemiology of Mycobacterium bovis infections in animals and man: A review. *International Journal of Tubercle and Lung Disease Journal*.

[8] Wedlock D. N., Skinner M. A., De Lisle G. W., Buddle B. M. (2002). Control of Mycobacterium bovis infections and the risk to human populations. *Microbes and Infection*.

[9] Jeon B.-Y., Je S., Park J. (2008). Variable number tandem repeat analysis of *Mycobacterium bovis* isolates from Gyeonggi-do, Korea. *Journal of Veterinary Science*.

[10] Gori A., Bandera A., Marchetti G. (2005). Spoligotyping and *Mycobacterium tuberculosis*. *Emerging Infectious Diseases*.

[11] Sola C., Filliol I., Legrand E., Mokrousov I., Rastogi N. (2001). *Mycobacterium tuberculosis* phylogeny reconstruction based on combined numerical analysis with IS1081, IS6110, VNTR, and DR-based spoligotyping suggests the existence of two new phylogeographical clades. *Journal of Molecular Evolution*.

[12] Filliol I., Driscoll J. R., Van Soolingen D. (2002). Global distribution of *Mycobacterium tuberculosis* spoligotypes. *Emerging Infectious Diseases*.

[13] Allix-Béguec C., Fauville-Dufaux M., Supply P. (2008). Three-year population-based evaluation of standardized mycobacterial interspersed repetitive-unit-variable-number tandem-repeat typing of *Mycobacterium tuberculosis*. *Journal of Clinical Microbiology*.

[14] Supply P., Lesjean S., Savine E., Kremer K., Van Soolingen D., Locht C. (2001). Automated high-throughput genotyping for study of global epidemiology of *Mycobacterium tuberculosis* based on mycobacterial interspersed repetitive units. *Journal of Clinical Microbiology*.

[15] Frothingham R., Meeker-O'Connell W. A. (1998). Genetic diversity in the *Mycobacterium tuberculosis* complex based on variable numbers of tandem DNA repeats. *Microbiology*.

[16] Mazars E., Lesjean S., Banuls A. L. (2001). High-resolution minisatellite-based typing as a portable approach to global analysis of *Mycobacterium tuberculosis* molecular epidemiology. *Proceedings of the National Academy of Sciences of the United States of America*.

[17] Goyal M., Young D., Zhang Y., Jenkins P. A., Shaw R. J. (1994). PCR amplification of variable sequence upstream of katG gene to subdivide strains of *Mycobacterium tuberculosis* complex. *Journal of Clinical Microbiology*.

[18] Namwat W., Luangsuk P., Palittapongarnpim P. (1998). The genetic diversity of *Mycobacterium tuberculosis* strains in thailand studied by amplification of DNA segments containing direct repetitive sequences. *The International Journal of Tuberculosis and Lung Disease*.

[19] Kremer K., van Soolingen D., Frothingham R. (1999). Comparison of methods based on different molecular epidemiological markers for typing of *Mycobacterium tuberculosis* complex strains: Interlaboratory study of discriminatory power and reproducibility. *Journal of Clinical Microbiology*.

[20] Hawkey P. M., Smith E. G., Evans J. T. (2003). Mycobacterial interspersed repetitive unit typing of *Mycobacterium tuberculosis* compared to IS6110-based restriction fragment length polymorphism analysis for investigation of apparently clustered cases of tuberculosis. *Journal of Clinical Microbiology*.

[21] Supply P. (2005). Multilocus Variable Number Tandem Repeat Genotyping of *Mycobacterium tuberculosis* Technical Guide. *Institut de Biologie/Institut Pasteur de Lille*.

[22] Brudey K., Driscoll J. R., Rigouts L. (2006). *Mycobacterium tuberculosis* complex genetic diversity: mining the fourth international spoligotyping database (SpolDB4) for classification, population genetics and epidemiology. *BMC Microbiology*.

[23] Van Dijk A. V. D. S., Makhoahle P. M., Rigouts L., Baba K. (2016). Diverse Molecular Genotypes of *Mycobacterium tuberculosis* Complex Isolates Circulating in the Free State, South Africa. *International Journal of Microbiology*.

[24] Warren R., Hauman J., Beyers N. (1996). Unexpectedly high strain diversity of *Mycobacterium tuberculosis* in a high incidence community. *South African Medical Journal*.

[25] Streicher E. M., Warren R. M., Kewley C. (2004). Genotypic and phenotypic characterization of drug-resistant *Mycobacterium tuberculosis* isolates from rural districts of the Western Cape Province of South Africa. *Journal of Clinical Microbiology*.

[26] Marais B. J., Victor T. C., Hesseling A. C. (2006). Beijing and Haarlem genotypes are overrepresented among children with drug-resistant tuberculosis in the Western Cape province of South Africa. *Journal of Clinical Microbiology*.

[27] Johnson R., Warren R. M., Van Der Spuy G. D. (2010). Drug-resistant tuberculosis epidemic in the Western Cape driven by a virulent Beijing genotype strain. *The International Journal of Tuberculosis and Lung Disease*.

[28] Green E., Obi L. C., Okoh A. I. (2013). IS6110 restriction fragment length polymorphism typing of drug-resistant *Mycobacterium tuberculosis* Strains from Northeast South Africa. *Journal of Health, Population and Nutrition*.

[29] Mlambo C. K., Warren R. M., Poswa X., Victor T. C., Duse A. G., Marais E. (2008). Genotypic diversity of extensively drug-resistant tuberculosis (XDR-TB) in South Africa. *The International Journal of Tuberculosis and Lung Disease*.

[30] Hove P., Molepo J., Dube S., Nchabeleng M. (2012). Genotypic diversity of *Mycobacterium tuberculosis* in Pretoria. *South African Journal of Epidemiology and Infection*.

[31] Marais B. J., Mlambo C. K., Rastogi N. (2013). Epidemic spread of multidrug-resistant tuberculosis in Johannesburg, South Africa. *Journal of Clinical Microbiology*.

[32] Pillay M., Sturm A. W. (2007). Evolution of the extensively drug-resistant F15/LAM4/KZN strain of *Mycobacterium tuberculosis* in KwaZulu-Natal, South Africa. *Clinical Infectious Diseases*.

[33] Gandhi N. R., Brust J. C. M., Moodley P. (2014). Minimal diversity of drug-resistant *Mycobacterium tuberculosis* strains, South Africa. *Emerging Infectious Diseases*.

[34] Bhembe N. L., Nwodo U. U., Okoh A. I. (2017). Clonality and genetic profiles of drug-resistant *Mycobacterium tuberculosis* in the Eastern Cape Province. *MicrobiologyOpen*.

[35] Berg S., Firdessa R., Habtamu M. (2009). The burden of mycobacterial disease in Ethiopian cattle: Implications for public health. *PLoS ONE*.

[36] Kamerbeek J., Schouls L., Kolk A. (1997). Simultaneous detection and strain differentiation of *Mycobacterium tuberculosis* for diagnosis and epidemiology. *Journal of Clinical Microbiology*.

[37] Demay C., Liens B., Burguière T. (2012). SITVITWEB—a publicly available international multimarker database for studying *Mycobacterium tuberculosis* genetic diversity and molecular epidemiology. *Infection, Genetics and Evolution*.

[38] Cadmus S., Hill V., van Soolingen D., Rastogi N. (2011). Spoligotype profile of *Mycobacterium tuberculosis* complex strains from HIV-positive and -negative patients in Nigeria: a comparative analysis. *Journal of Clinical Microbiology*.

[39] Shabbeer A., Cowan L. S., Ozcaglar C. (2012). TB-Lineage: an online tool for classification and analysis of strains of *Mycobacterium tuberculosis* complex. *Infection, Genetics and Evolution*.

[40] Gagneux S., Small P. M. (2007). Global phylogeography of *Mycobacterium tuberculosis* and implications for tuberculosis product development. *The Lancet Infectious Diseases*.

[41] Weniger T., Krawczyk J., Supply P., Niemann S., Harmsen D. (2010). MIRU-VNTRplus: A web tool for polyphasic genotyping of *Mycobacterium tuberculosis* complex bacteria. *Nucleic Acids Research*.

[42] Hunter P. R., Gaston M. A. (1988). Numerical index of the discriminatory ability of typing systems: an application of Simpson's index of diversity. *Journal of Clinical Microbiology*.

[43] TB Facts.org TB Statistics for South Africa–National & provincial. https://www.tbfacts.org/tb-statistics-south-africa/.

[44] Mabunda T. E., Ramalivhana N. J., Dambisya Y. M. (2014). Mortality associated with tuberculosis/HIV co-infection among patients on TB treatment in the Limpopo province, South Africa. *African Health Sciences*.

[45] Van Dijk A. V. D. S., Makhoahle P. M., Rigouts L., Baba K. (2016). Diverse Molecular Genotypes of *Mycobacterium tuberculosis* Complex Isolates Circulating in the Free State, South Africa. *International Journal of Microbiology*.

[46] Said H. M., Kock M. M., Ismail N. A. (2012). Molecular characterization and second-line antituberculosis drug resistance patterns of multidrug-resistant *Mycobacterium tuberculosis* Isolates from the northern region of South Africa. *Journal of Clinical Microbiology*.

[47] Stavrum R., Mphahlele M., Ovreas K. (2009). High diversity of *Mycobacterium tuberculosis* genotypes in South Africa and preponderance of mixed infections among ST53 isolates. *Journal of Clinical Microbiology*.

[48] Roring S., Scott A. N., Hewinson R. G., Neill S. D., Skuce R. A. (2004). Evaluation of variable number tandem repeat (VNTR) loci in molecular typing of Mycobacterium bovis isolates from Ireland. *Veterinary Microbiology*.

[49] Hilty M., Diguimbaye C., Schelling E., Baggi F., Tanner M., Zinsstag J. (2005). Evaluation of the discriminatory power of variable number tandem repeat (VNTR) typing of Mycobacterium bovis strains. *Veterinary Microbiology*.

[50] Gagneux S., DeRiemer K., Van T. (2006). Variable host-pathogen compatibility in *Mycobacterium tuberculosis*. *Proceedings of National Academy of Science of the United States of America*.

[51] Streicher E. M., Victor T. C., Van Der Spuy G. (2007). Spoligotype signatures in the *Mycobacterium tuberculosis* complex. *Journal of Clinical Microbiology*.

[52] Gutierrez M. C., Ahmed N., Willery E. (2006). Predominance of ancestral lineages of *Mycobacterium tuberculosis* in India. *Emerging Infectious Diseases*.

[53] Asiimwe B. B., Ghebremichael S., Kallenius G., Koivula T., Joloba M. L. (2008). *Mycobacterium tuberculosis* spoligotypes and drug susceptibility pattern of isolates from tuberculosis patients in peri-urban Kampala, Uganda. *BMC Infectious Diseases*.

[54] Phyu S., Stavrum R., Lwin T., Svendsen S. S., Ti T., Grewal H. M. S. (2009). Predominance of *Mycobacterium tuberculosis* EAI and Beijing lineages in Yangon, Myanmar. *Journal of Clinical Microbiology*.

[55] Victor T. C., de Haas P. E. W., Jordaan A. M. (2004). Molecular characteristics and global spread of *Mycobacterium tuberculosis* with a Western Cape F11 genotype. *Journal of Clinical Microbiology*.

[56] Mardassi H., Namouchi A., Haltiti R. (2005). Tuberculosis due to resistant Haarlem strain, Tunisia. *Emerging Infectious Diseases*.

[57] Namouchi A., Karboul A., Mhenni B. (2008). Genetic profiling of *Mycobacterium tuberculosis* in Tunisia: Predominance and evidence for the establishment of a few genotypes. *Journal of Medical Microbiology*.

[58] Chihota V., Apers L., Mungofa S. (2007). Predominance of a single genotype of *Mycobacterium tuberculosis* in regions of Southern Africa. *The International Journal of Tuberculosis and Lung Disease*.

[59] Todar K. (2008). Tuberculosis. *Todar’s Online Textbook of Bacteriology*.

[60] Miller M. A. Tuberculosis in South African wildlife: why is it important? naugural lecture. http://www.sun.ac.za/english/Inaugurallectures/Inaugural%20lectures/InauguralLectureProfMiller.pdf.

[61] Bifani P. J., Mathema B., Kurepina N. E., Kreiswirth B. N. (2002). Global dissemination of the *Mycobacterium tuberculosis* W-Beijing family strains. *Trends in Microbiology*.

[62] Toungoussova O. S., Caugant D. A., Sandven P., Mariandyshev A. O., Bjune G. (2004). Impact of drug resistance on fitness of *Mycobacterium tuberculosis* strains of the W-Beijing genotype. *FEMS Immunology & Medical Microbiology*.

[63] Almeida D., Rodrigues C., Ashavaid T. F., Lalvani A., Udwadia Z. F., Mehta A. (2005). High incidence of the beijing genotype among multidrug-resistant isolates of *Mycobacterium tuberculosis* in a Tertiary Care Center in Mumbai, India. *Clinical Infectious Diseases*.

[64] Kibiki G. S., Mulder B., Dolmans W. M. V. (2007). M. tuberculosis genotypic diversity and drug susceptibility pattern in HIV- infected and non-HIV-infected patients in northern Tanzania. *BMC Microbiology*.

[65] Homolka S., Post E., Oberhauser B. (2008). High genetic diversity among *Mycobacterium tuberculosis* complex strains from Sierra Leone. *BMC Microbiology*.

[66] Piercy J., Werling D., Coffey T. J. (2007). Differential responses of bovine macrophages to infection with bovine-specific and non-bovine specific mycobacteria. *Tuberculosis*.

[67] Hackendahl N. C., Mawby D. I., Bemis D. A., Beazley S. L. (2004). Putative transmission of *Mycobacterium tuberculosis* infection from a human to a dog. *Journal of the American Veterinary Medical Association*.

[68] Prasad H. K., Singhal A., Mishra A. (2009). High diversity of *Mycobacterium tuberculosis* genotypes in South Africa and preponderance of mixed infections among ST53 isolates. *Journal of Clinical Microbiology*.

[69] Davies P. D. O. (2006). Tuberculosis in humans and animals: Are we a threat to each other?. *Journal of the Royal Society of Medicine*.

[70] Chen Y., Chao Y., Deng Q. (2009). Potential challenges to the Stop TB Plan for humans in China; cattle maintain M. bovis and M. tuberculosis. *Tuberculosis*.

[71] Rodwell T. C., Moore M., Moser K. S., Brodine S. K., Strathdee S. A. (2008). Tuberculosis from Mycobacterium bovis in binational communities, United States. *Emerging Infectious Diseases*.

[72] Du Y., Qi Y., Yu L. (2011). Molecular characterization of *Mycobacterium tuberculosis* complex (MTBC) isolated from cattle in northeast and northwest China. *Research in Veterinary Science*.

[73] Ocepek M., Pate M., Žolnir-Dovč M., Poljak M. (2005). Transmission of *Mycobacterium tuberculosis* from human to cattle. *Journal of Clinical Microbiology*.

[74] Fetene T., Kebede N., Alem G. (2011). Tuberculosis infection in animal and human populations in three districts of Western Gojam, Ethiopia. *Zoonoses and Public Health*.

[75] Romero B., Rodríguez S., Bezos J. (2011). Humans as source of *Mycobacterium tuberculosis* infection in cattle, Spain. *Emerging Infectious Diseases*.

[76] World Health Organization (WHO) Global Tuberculosis Report. http://apps.who.int/iris/bitstream/10665/250441/1/9789241565394-eng.pdf?ua=1.

[77] Van Soolingen D. (2001). Molecular epidemiology of tuberculosis and other mycobacterial infections: main methodologies and achievements. *Journal of Internal Medicine*.

[78] Glynn J. R., Bauer J., de Boer A. S. (1999). Interpreting DNA fingerprint clusters of *Mycobacterium tuberculosis*. *International Journal of Tuberculosis and Lung Diseases*.

[79] Braden C. R., Templeton G. L., Donald Cave M. (1997). Interpretation of restriction fragment length polymorphism analysis of *Mycobacterium tuberculosis* isolates from a state with a large rural population. *The Journal of Infectious Diseases*.

[80] Lockman S., Sheppard J. D., Braden C. R. (2001). Molecular and conventional epidemiology of *Mycobacterium tuberculosis* in Botswana: A population-based prospective study of 301 pulmonary tuberculosis patients. *Journal of Clinical Microbiology*.

[81] Godfrey-Faussett P., Sonnenberg P., Shearer S. C. (2000). Tuberculosis control and molecular epidemiology in a South African gold-mining community. *The Lancet*.

[82] Verver S., Warren R. M., Munch Z. (2004). Transmission of tuberculosis in a high incidence urban community in South Africa. *International Journal of Epidemiology*.

